# Saphenous vein grafts from thigh versus calf: Comparison of angiographic patency and clinical outcomes

**DOI:** 10.1016/j.xjon.2026.102079

**Published:** 2026-08-22

**Authors:** Suk Ho Sohn, Hee Ju Hong, Suji Oh, Yuri Lee, Yoonjin Kang, Jae Woong Choi, Ho Young Hwang

**Affiliations:** Department of Thoracic and Cardiovascular Surgery, Seoul National University Hospital, Seoul National University College of Medicine, Seoul, Republic of Korea

**Keywords:** coronary artery bypass grafting, saphenous vein, angiography, outcomes

## Abstract

**Objectives:**

This study compared angiographic patency and clinical outcomes between patients who received saphenous vein (SV) grafts from the upper leg (UL) and those who received grafts from the lower leg (LL) for coronary artery bypass grafting (CABG).

**Methods:**

A total of 1630 patients who received primary isolated off-pump CABG using the SV harvested via the no-touch technique from the UL (UL group, n = 203) or the LL (LL group, n = 1427) were enrolled in this study. Propensity score matching was performed, and 202 pairs were matched. Early, 1-year, and 5-year graft angiograms were obtained for 99.5%, 85.7%, and 31.2% of the cohort, respectively. The median follow-up duration was 85.8 months. The primary outcome was midterm angiographic patency, and the secondary outcomes were midterm clinical outcomes.

**Results:**

There was no significant difference in baseline characteristics between the matched groups. Early clinical outcomes, including operative mortality (0.8%, 13 out of 1630), demonstrated no intergroup differences. Angiographic patency for SV grafts was not significantly different at early (98.3% vs 98.5% in the UL group vs the LL group, *P* = .80), 1-year (94.8% vs 95.4% in the UL group vs the LL group, *P* = .74), or 5-year evaluation (97.8% vs 96.0% in the UL group vs the LL group, *P* = .43). Midterm survival up to 7 years was comparable between the groups (*P* = .81).

**Conclusions:**

SV grafts from the UL demonstrated comparable 5-year angiographic patency and 7-year clinical outcomes compared with those from the LL during off-pump CABG.

## Graphical Abstract

**Figure:**
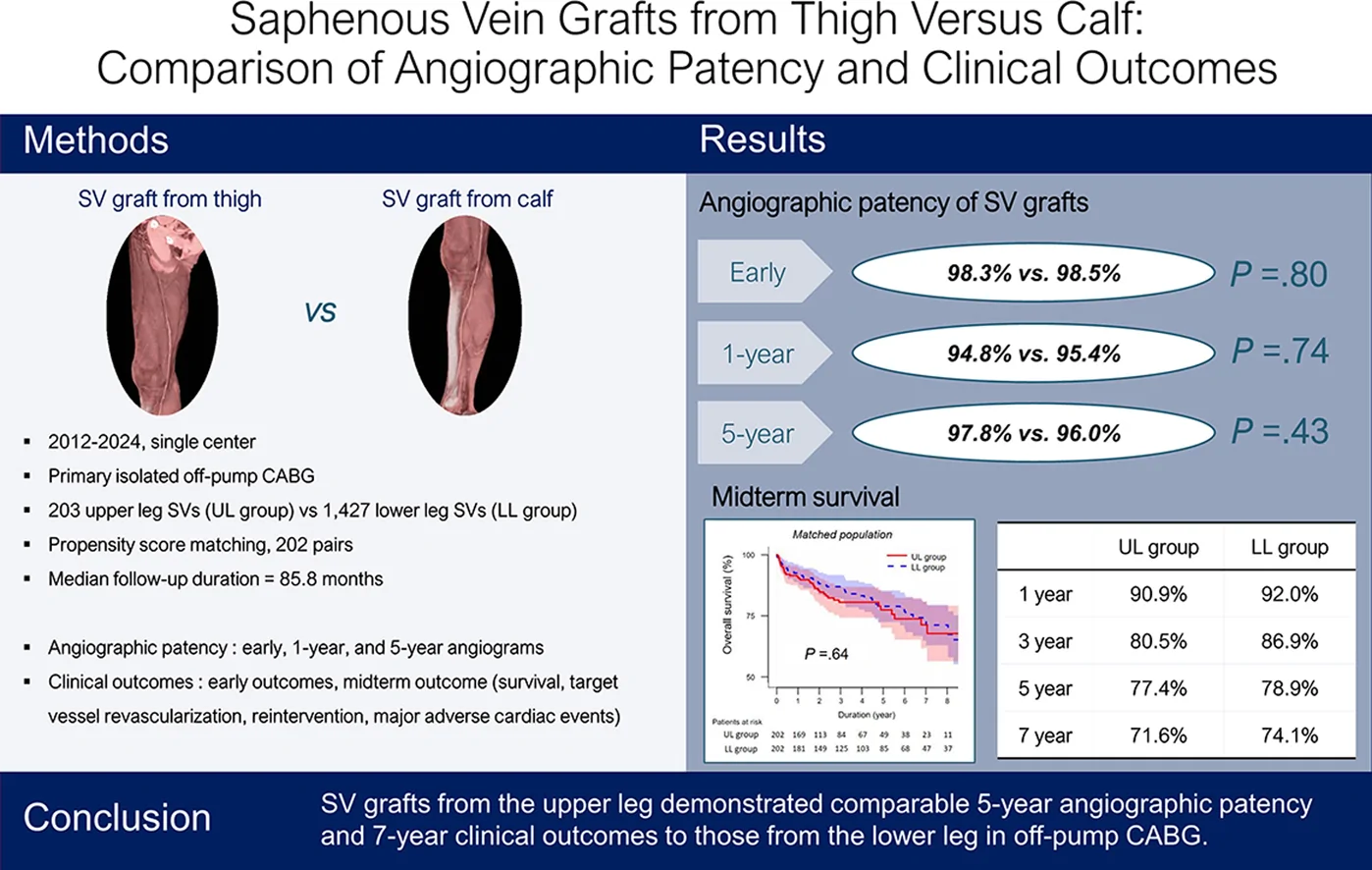

**Figure:**
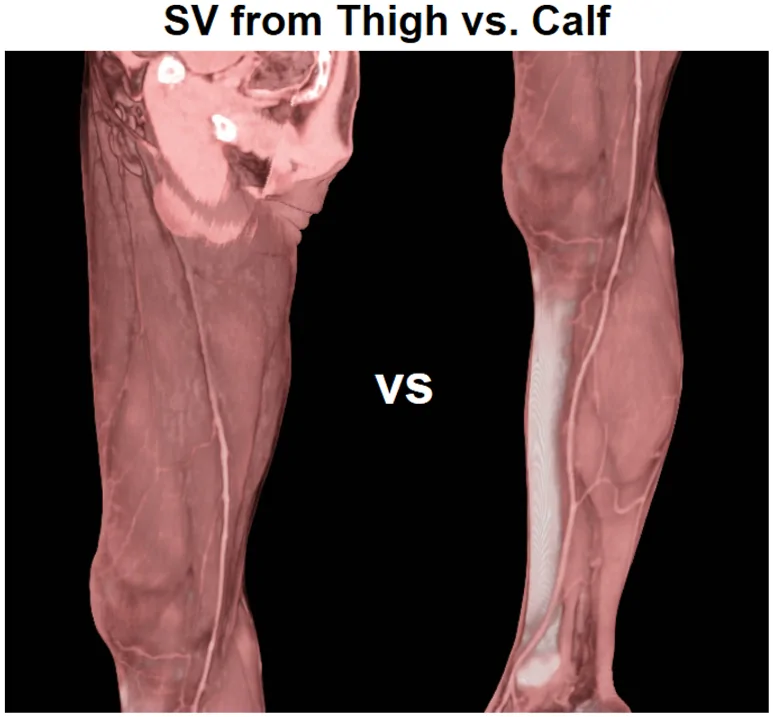
No-touch saphenous vein grafts from thigh versus calf.

**Central Message**

The no-touch saphenous vein grafts harvested from the upper leg demonstrated comparable 5-year angiographic patency and 7-year clinical outcomes compared with those harvested from the lower leg.

**Perspective**

Due to the size discrepancy in the target vessels or in the composite internal thoracic artery grafts used for revascularization, there have been concerns that saphenous vein grafts from the upper leg would show inferior outcomes compared with those from the lower leg. This study revealed that saphenous vein grafts from the upper leg demonstrated comparable midterm angiographic patency and clinical outcomes compared with those from the lower leg.

The internal thoracic artery (ITA) is widely regarded as the conduit of choice for coronary artery bypass grafting (CABG) owing to its excellent long-term patency and associated survival benefit.^1^ With respect to the second conduit, multiple arterial grafting with an additional arterial graft has been increasingly advocated and supported by numerous reports.^2^^,^^3^ Nevertheless, its adoption remains limited in contemporary practice, accounting for only 3.9% to 34.2% of CABG procedures in North America and Europe.^4^ Consequently, saphenous vein (SV) grafts continue to be most frequently utilized conduits worldwide despite their well-documented susceptibility to early and late graft failure.^5^^,^^6^

Recently, the no-touch (NT) SV harvesting technique—characterized by minimal manipulation of the vein and avoidance of manual intraluminal distension—has been introduced as a strategy to improve graft durability.^7^ Accumulating evidence suggests that compared with conventionally harvested SV grafts, NT-SV grafts demonstrate superior short- and long-term patency,^8^, ^9^, ^10^ with some studies reporting outcomes comparable with those of arterial grafts.^11^, ^12^, ^13^

In routine clinical practice, SV grafts from the thigh (upper leg [UL]) are more frequently used than those from the calf (lower leg [LL]) for CABG. However, whether the harvest site influences angiographic patency or long-term clinical outcomes remains poorly understood. Despite the widespread preference for SV grafts from the UL, CABG using SV grafts from the LL also demonstrated excellent outcomes comparable with multiarterial grafting in terms of angiographic patency and long-term clinical outcomes in previous prospective randomized and large retrospective studies.^12^^,^^13^ The impact of SV harvest location has not been systematically examined in large clinical cohorts. Therefore, the present study aimed to compare angiographic graft patency and long-term clinical outcomes in patients who underwent CABG with SV grafts harvested from the UL versus the LL.

## Materials and Methods

### Patient Selection

The study protocol was reviewed by the institutional review board and approved as a minimal-risk retrospective study (approval number H-2601-169-1713), and individual informed consent was waived. Among the 2212 patients who underwent CABG from 2012 to 2024, after those with concomitant CABG (n = 252), redo CABG (n = 69), and on-pump CABG (n = 62) were excluded, a total of 1829 patients who underwent primary isolated off-pump CABG were included. Among these patients, 1630 patients who used a single SV graft from either the UL (UL group, n = 203) or the LL (LL group, n = 1427) were ultimately enrolled in the study (Figure 1).

**Figure 1:**
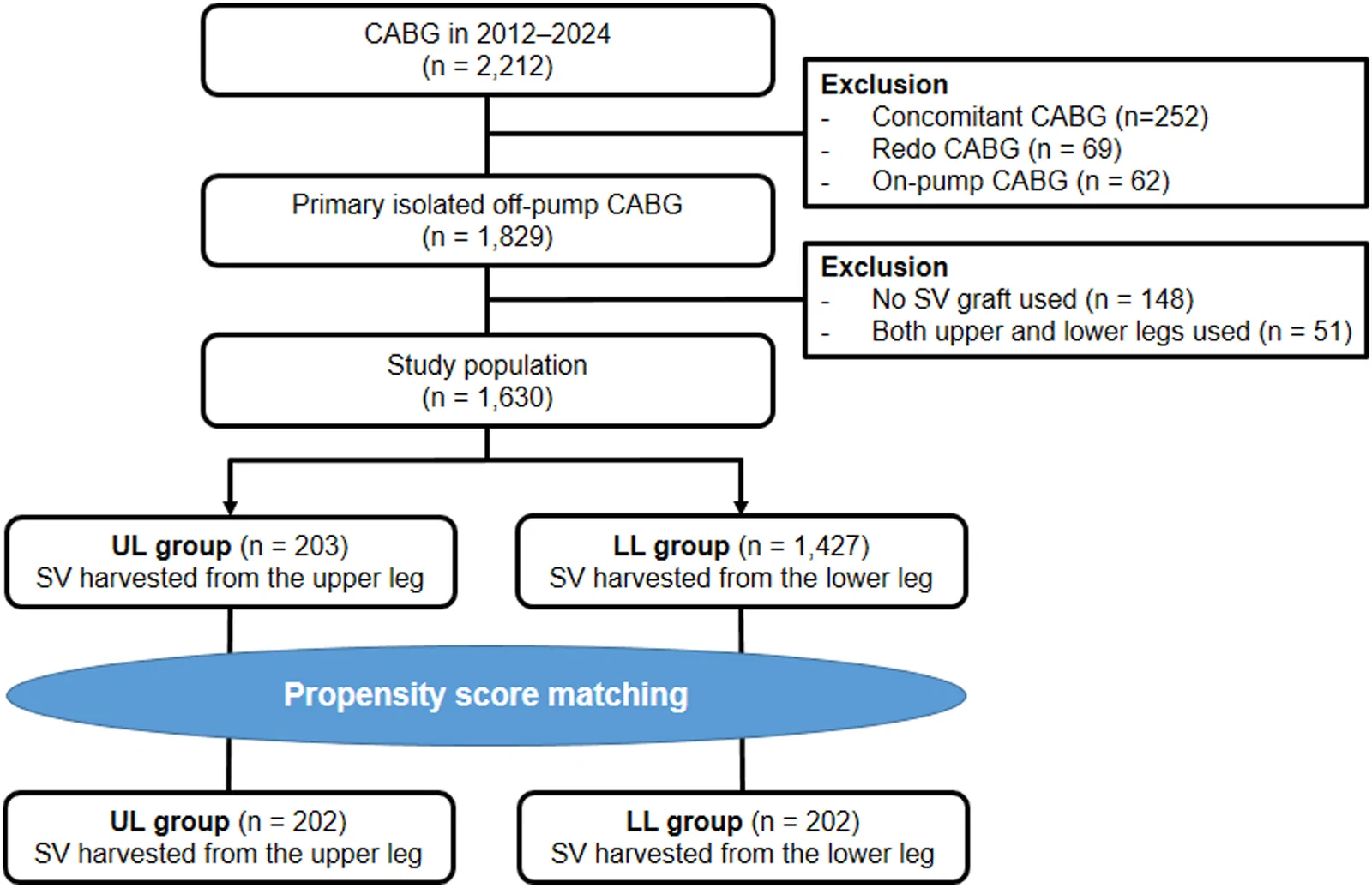
Flow diagram of patient enrollment. *CABG*, Coronary artery bypass grafting; *LL*, lower leg; *SV*, saphenous vein; *UL*, upper leg.

### Operative Techniques and Revascularization Strategy

The basic surgical procedures and strategies of CABG have been described previously.^12^^,^^14^ During the study period, off-pump CABG using Y-composite grafting based on the in situ left internal thoracic artery (LITA) was the preferred revascularization strategy at our institution. The SV has been used as the preferred second conduit to construct Y-composite grafts. However, sequential grafting with I-composite grafts based on the in situ LITA was used instead in some patients in whom use of the LITA as an inflow source was not suitable, and the right internal thoracic artery (RITA) served as an alternative inflow source to construct Y- or I-composite grafts based on the in situ RITA. Aortocoronary SV grafting was also performed in some patients.

During the study period, the NT-SV was harvested preferentially from the LL to alleviate possible size mismatch with native coronary arteries or ITAs. In patients whose SV from the LL had a very small caliber, SV from the UL was alternatively chosen. The NT technique was also used for harvesting UL SV grafts. The reversed SV was anastomosed to the LITA, RITA, or ascending aorta in an end-to-side fashion immediately after the harvest without using a storage solution. After the SV graft anastomosis was complete, the left anterior descending coronary artery territory was revascularized first whenever possible. The left circumflex coronary artery territory was then grafted and followed by the right coronary artery territory revascularization. A sequential anastomotic technique was used as needed. The 2 surgeons involved in this study used identical operative strategies regarding the selection of SV harvest location, and there was no difference in the proportion of lower-leg SVs between the two surgeons (Table E1).

Patients were treated with dual antiplatelet therapy until 1 year postoperatively, and single antiplatelet therapy thereafter. All patients also received statins.

### Evaluation of Clinical Outcomes

Operative mortality was defined as any death within 30 days after surgery or during the same hospital admission. After discharge, all patients underwent regular postoperative follow-up at the outpatient clinic at 3- to 6-month intervals and were interviewed by telephone to confirm their condition if the last clinic visit was missed. For patients who were lost to follow-up, national health insurance data and information from Statistics Korea, a central organization for statistics under the Ministry of Strategy and Finance, were utilized to confirm their survival or date of death. The clinical follow-up was closed on December 31, 2025. The follow-up data were complete for 100.0% of overall survival, with a median follow-up duration of 85.8 months (interquartile range [IQR], 66.8, 108.9).

SV harvest site complication was defined as a leg wound dehiscence or infection requiring medical or surgical intervention. Target vessel revascularization (TVR) was defined as percutaneous or surgical coronary interventions on target vessels that were bypassed during the index operation, and non-TVR was defined as revascularization for a new lesion outside the target vessels. Reintervention included both TVRs and non-TVRs. Major adverse cardiac events (MACEs) included cardiac death, acute myocardial infarction, and coronary reintervention.

### Angiographic Evaluation of Graft Patency

Early, 1-year, and 5-year follow-up coronary angiograms were performed with the patient’s consent, regardless of the presence of angina symptoms.^15^ Patients who died, refused angiographic evaluation, or had renal function impairment but who were not dependent on renal replacement therapy were excluded from angiographic follow-up (Table E2). Early postoperative (1 day [IQR, 1, 1]) graft evaluation was performed in 99.5% (1622 out of 1630) of the study patients with conventional angiography. One-year (12.5 months [IQR, 12.0, 13.1]) and 5-year (60.4 months [IQR, 59.7, 62.2]) angiograms were performed for 85.7% (1404 out of 1630) and 31.2% (508 out of 1630) of the study population, respectively. Multidetector computed tomography angiography was performed instead of conventional angiography in 63.2% (887 out of 1404) and 85.8% (436 out of 508) of the patients for the 1- and 5-year graft evaluations, respectively (Table E3). The graft patency was assessed using standardized classification described by FitzGibbon and colleagues.^16^

### Statistical Analysis

Statistical analysis was performed using IBM SPSS version 25.0 (IBM Corp.) and SAS version 9.4 (SAS Institute Inc.). Continuous variables are presented as the means ± standard deviations or medians with IQRs, and categorical variables are presented as the numbers and proportions. Propensity score matching was performed to adjust for differences in baseline characteristics between the groups. The propensity scores were estimated with all 18 preoperative variables shown in Table 1. The nearest neighborhood (greedy matching) within a caliper width of 0.2 of the standard deviation of the logit of the propensity scores was used to match the patients at a ratio of 1:1. The balance of covariates was evaluated with standardized mean differences (SMDs). An absolute SMD ≤0.1 was considered to indicate a negligible difference between the groups.

**Table 1:** Preoperative characteristics and risk factors

Variables	Entire population				Matched population		
	UL group (n = 203)	LL group (n = 1427)	SMD	*P*	UL group (n = 202)	LL group (n = 202)	SMD
Age, y	68.5 ± 11.0	66.5 ± 10.1	0.190	<.001	68.5 ± 11.0	68.8 ± 10.0	−0.030
Sex, female, n (%)	84 (41.1)	289 (20.3)	0.470	<.001	83 (41.1)	76 (37.6)	0.080
Risk factors, n (%)							
Smoking	78 (38.4)	602 (42.2)	0.077	.35	78 (38.6)	85 (42.1)	0.070
Body mass index	23.3 ± 3.2	24.7 ± 3.3	−0.433	<.001	23.3 ± 3.2	23.2 ± 3.5	0.040
Hypertension	148 (72.9)	994 (69.7)	−0.072	.39	147 (72.8)	150 (74.3)	0.030
Diabetes mellitus	124 (61.1)	754 (52.8)	−0.167	.03	123 (60.9)	117 (57.9)	−0.060
Dyslipidemia	78 (38.4)	627 (43.9)	0.112	.16	77 (38.1)	72 (35.6)	−0.050
History of stroke	42 (20.7)	180 (12.6)	−0.218	.002	42 (20.8)	45 (22.3)	0.040
Dialysis	24 (11.8)	111 (7.8)	−0.136	.07	23 (11.4)	19 (9.4)	−0.070
Chronic obstructive pulmonary disease	12 (5.9)	68 (4.8)	−0.051	.59	12 (5.9)	16 (7.9)	0.090
Atrial fibrillation	3 (1.5)	29 (2.0)	0.042	.79	3 (1.5)	3 (1.5)	<0.001
Peripheral vascular disease	26 (12.8)	133 (9.3)	−0.111	.15	26 (12.9)	29 (14.4)	0.050
Previous percutaneous coronary intervention	48 (23.6)	317 (22.2)	−0.034	.71	47 (23.3)	42 (20.8)	−0.060
Left ventricular ejection fraction ≤0.35	32 (15.8)	168 (11.8)	−0.116	.13	32 (15.8)	31 (15.3)	−0.010
Acute coronary syndrome	131 (64.5)	854 (59.8)	−0.097	.23	130 (64.4)	126 (62.4)	−0.040
Three vessel disease, n (%)	152 (74.9)	1062 (74.4)	−0.010	.96	151 (74.8)	145 (71.8)	−0.070
Left main disease, n (%)	69 (34.0)	509 (35.7)	0.035	.70	69 (34.2)	72 (35.6)	0.030
Emergency operation, n (%)	26 (12.8)	108 (7.6)	−0.174	.02	25 (12.4)	23 (11.4)	−0.030

Continuous variables are presented as mean ± standard deviation for normally distributed variables. *LL*, Lower leg; *SMD*, standardized mean difference; *UL*, upper leg.

Comparisons of operative details and early clinical outcomes between the two groups were performed using the chi-square test or Fisher exact test for categorical variables and the Student *t* test or Wilcoxon rank sum test for continuous variables. For comparison between the matched groups, the McNemar test, paired Student *t* test, and Wilcoxon signed-rank test were used. Graft patency was compared using logistic regression at each time point, and generalized estimating equations accounted for paired measurements in the matched population. When sparse events were observed, Firth’s bias reduction method was used.^17^^,^^18^

For longitudinal data analyses, overall survival was estimated using the Kaplan-Meier method, and the hazard ratio was estimated using a proportional hazards model. TVR, reintervention, and MACEs were analyzed with cumulative incidence curves, with death considered as a competing event. Comparison of cumulative incidence curves between the 2 groups was performed using a subdistribution hazards model.^19^ A *P* value of <.05 was considered to indicate statistical significance.

## Results

### Patient Characteristics

The UL group contained more patients who were older, female, had a lower body mass index, and had more comorbidities. However, these differences became nonsignificant after matching (Table 1).

### Operative Data

In terms of SV grafting strategy, LITA-based Y-composite grafting was the most frequently used, followed by LITA-based I-composite, aortocoronary, RITA-based I-composite, and RITA-based Y-composite grafting.

In the matched population, the average number of distal anastomoses per patient was comparable between the groups (3.55 ± 0.89 in the UL group vs 3.66 ± 0.93 in the LL group, *P* = .21). The average numbers of distal anastomoses per SV and per territory were also comparable between the groups (Table E4).

### Early and Long-Term Clinical Outcomes

The operative mortality was 0.8% (13 out of 1630 patients). In the matched population, there were no differences in early postoperative outcomes between the groups. SV harvest site complications also showed no significant differences between groups (2.0% in the UL group vs 2.0% in the LL group, *P* ≥ .99) (Table 2).

**Table 2:** Comparison of early clinical outcomes

Variables	Entire population			Matched population		
	UL group (n = 203)	LL group (n = 1427)	*P*	UL group (n = 202)	LL group (n = 202)	*P*
Operative mortality, n (%)	4 (2.0)	9 (0.6)	.07	3 (1.5)	2 (1.0)	.66
Postoperative complications, n (%)						
Postoperative atrial fibrillation	68 (33.5)	450 (31.5)	.57	68 (33.7)	75 (37.1)	.47
Acute kidney injury	11 (5.4)	67 (4.7)	.65	11 (5.4)	15 (7.4)	.41
Respiratory complications	17 (8.4)	82 (5.7)	.14	17 (8.4)	19 (9.4)	.74
Delirium	16 (7.9)	66 (4.6)	.05	16 (7.9)	9 (4.5)	.14
Bleeding reoperation	2 (1.0)	17 (1.2)	>.99	2 (1.0)	2 (1.0)	>.99
Stroke	0 (0.0)	5 (0.4)	>.99	0 (0.0)	2 (1.0)	.50
Deep sternal wound infection	2 (1.0)	4 (0.3)	.17	2 (1.0)	0 (0.0)	.50
SV harvest site complications	4 (2.0)	25 (1.8)	.78	4 (2.0)	4 (2.0)	>.99

*LL*, Lower leg; *SV*, saphenous vein; *UL*, upper leg.

In the entire population, the 7-year overall survival rate was higher in the LL group (Figure E1). In the matched population, however, it was not significantly different between the groups. The 7-year cumulative incidences of TVR, reintervention, and MACEs were also comparable between the groups in the matched population (Figure 2).

**Figure 2:**
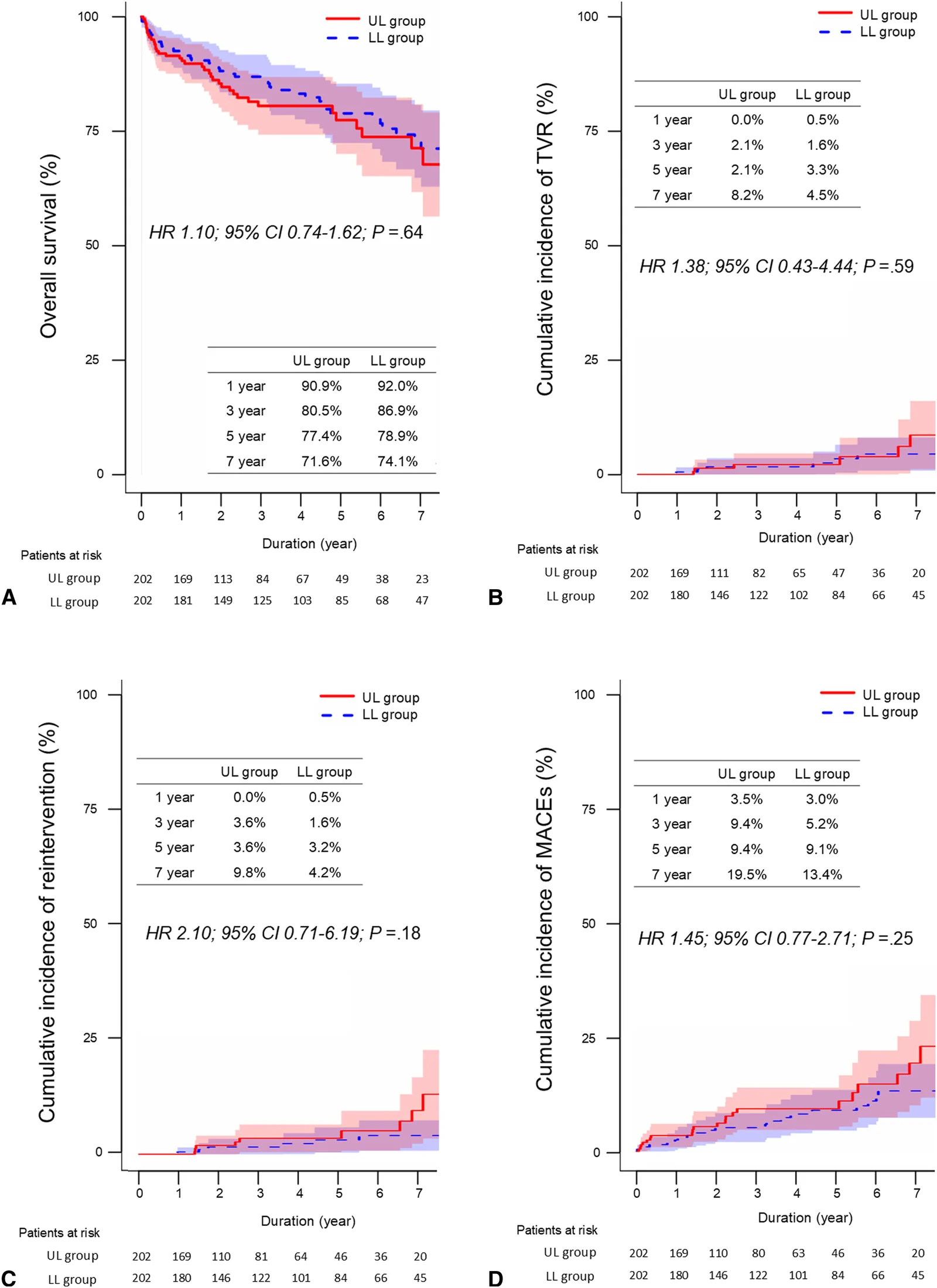
Comparison of (A) overall survival, (B) cumulative incidence of TVR, (C) cumulative incidence of reintervention, and (D) cumulative incidence of MACEs between groups in the matched population. Shading indicates the 95% CI. *HR*, Hazard ratio; *LL*, lower leg; *MACE*, major adverse cardiac event; *TVR*, target vessel revascularization; *UL*, upper leg.

### Angiographic Graft Patency

In the matched population, early, 1-year, and 5-year overall patency rates were comparable between the groups. When confined to the patency of the SV grafts, early, 1-year, and 5-year patency rates were also comparable between the groups (Table E5).

Subgroup analysis of revascularized territories revealed no significant differences in early, 1-year, or 5-year patency. In the subgroup analysis according to the position within sequential anastomoses in an SV graft, no significant differences between the groups were observed for anastomoses at both sequential and terminal positions (Table 3).

**Table 3:** Comparison of early, 1-year, and 5-year angiographic patency rates of the anastomoses with SV grafts

Variables	Entire population			Matched population		
	UL group (n = 203)	LL group (n = 1427)	*P*	UL group (n = 202)	LL group (n = 202)	*P*
Early angiography	(n = 201)	(n = 1421)		(n = 201)	(n = 200)	
Overall SV anastomoses	98.3% (526/535)	98.0% (3790/3867)	.624	98.3% (526/535)	98.5% (531/539)	.795
Anastomosed to LAD territory	100.0% (166/166)	99.8% (1093/1095)	.861	100.0% (166/166)	100.0% (153/153)	-
Anastomosed to LCX territory	98.5% (192/195)	98.9% (1502/1519)	.608	98.5% (192/195)	99.5% (208/209)	.311
Anastomosed to RCA territory	96.6% (168/174)	95.4% (1195/1253)	.486	96.6% (168/174)	96.0% (170/177)	.805
As sequential anastomosis	99.7% (329/330)	99.5% (2397/2409)	.634	99.7% (329/330)	99.7% (332/333)	.995
As terminal anastomosis	96.1% (197/205)	95.5% (1393/1458)	.717	96.1% (197/205)	96.6% (199/206)	.789
1-y angiography	(n = 158)	(n = 1246)		(n = 158)	(n = 166)	
Overall SV anastomoses	94.8% (399/421)	94.9% (3245/3419)	.916	94.8% (399/421)	95.4% (431/452)	.736
Anastomosed to LAD territory	98.4% (125/127)	98.2% (941/958)	.872	98.4% (125/127)	97.5% (119/122)	.616
Anastomosed to LCX territory	94.9% (149/157)	97.1% (1306/1345)	.143	94.9% (149/157)	97.8% (176/180)	.177
Anastomosed to RCA territory	91.2% (125/137)	89.4% (998/1116)	.517	91.2% (125/137)	90.7% (136/150)	.873
As sequential anastomosis	96.9% (251/259)	98.2% (2106/2144)	.153	96.9% (251/259)	97.9% (276/282)	.491
As terminal anastomosis	91.4% (148/162)	89.3% (1139/1275)	.429	91.4% (148/162)	91.2% (155/170)	.955
5-y angiography	(n = 36)	(n = 472)		(n = 36)	(n = 67)	
Overall SV anastomoses	97.8% (91/93)	93.7% (1154/1231)	.118	97.8% (91/93)	96.0% (169/176)	.425
Anastomosed to LAD territory	100.0% (27/27)	98.2% (328/334)	.955	100.0% (27/27)	98.0% (49/50)	.250
Anastomosed to LCX territory	97.4% (37/38)	94.5% (484/512)	.464	97.4% (37/38)	95.7% (67/70)	.667
Anastomosed to RCA territory	96.4% (27/28)	88.8% (342/385)	.237	96.4% (27/28)	94.6% (53/56)	.720
As sequential anastomosis	98.2% (54/55)	97.1% (724/746)	.629	98.2% (54/55)	98.1% (104/106)	.976
As terminal anastomosis	97.4% (37/38)	88.7% (430/485)	.129	97.4% (37/38)	92.9% (65/70)	.347

*LAD*, Left anterior descending; *LCX*, left circumflex; *LL*, lower leg; *RCA*, right coronary artery; *SV*, saphenous vein; *UL*, upper leg.

### Multivariable Analysis for Long-Term Outcomes

Multivariable analyses demonstrated that SV harvest location was not associated with long-term outcomes, including overall survival, TVR, reintervention, and MACEs (Table E6, Table E7, Table E8, Table E9).

## Discussion

The principal finding of this study is that the harvest site of SV—UL versus LL—was not associated with differences in 5-year angiographic patency or 7-year clinical outcomes following CABG (Figure 3).

**Figure 3:**
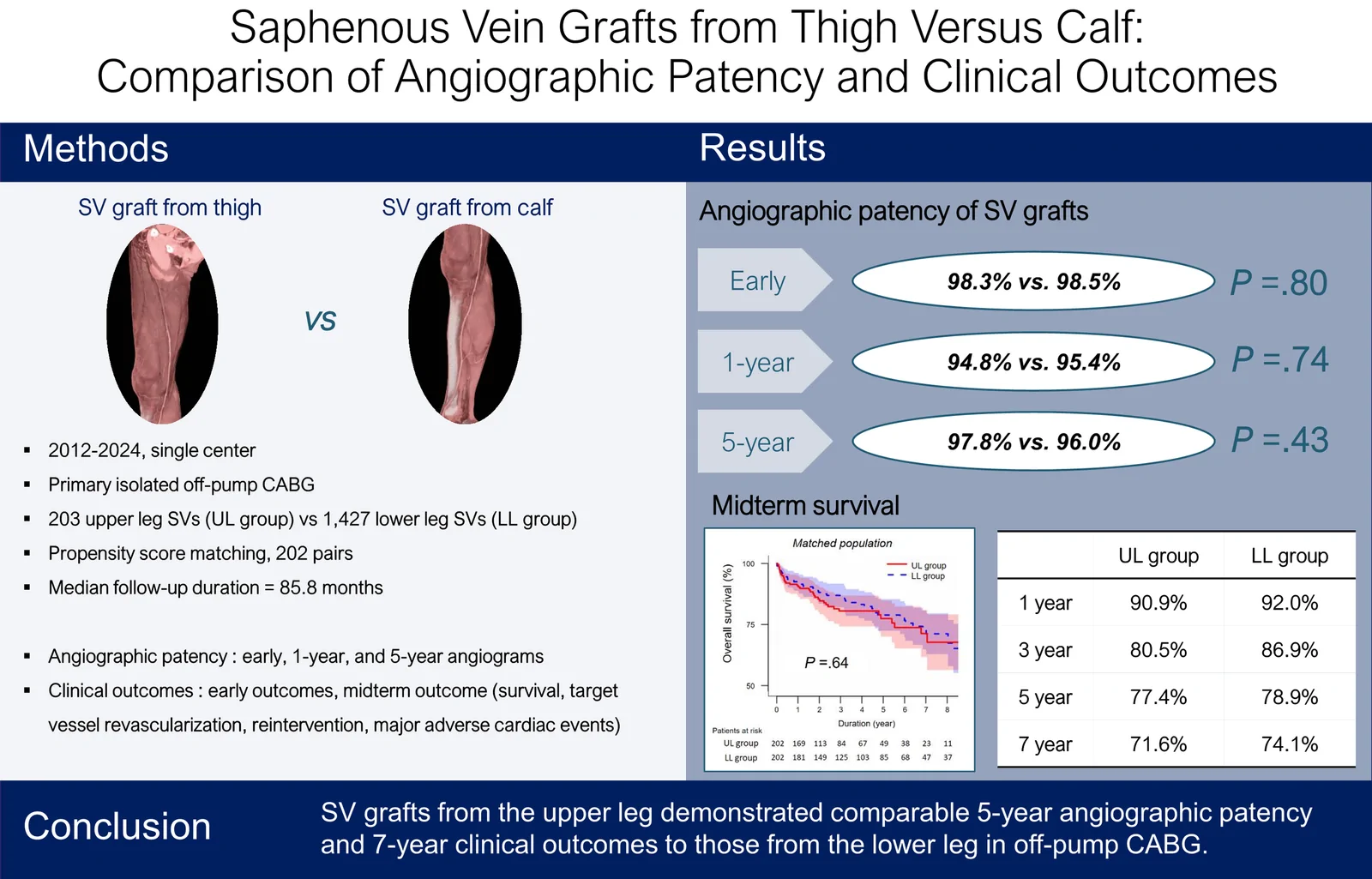
Overall 1630 patients who received primary isolated off-pump CABG using SV from the upper leg or the lower leg were enrolled in this study. Propensity score matching was performed, and 202 pairs were compared. SV grafts harvested from the upper leg demonstrated comparable 5-year angiographic patency and 7-year clinical outcomes to those harvested from the lower leg. *CABG*, Coronary artery bypass grafting; *LL*, lower leg; *SV*, saphenous vein; *UL*, upper leg.

Although numerous studies have compared SV grafts with arterial conduits,^9^^,^^12^^,^^13^^,^^20^, ^21^, ^22^, ^23^ the influence of harvest location has rarely been examined as a potential determinant of graft durability. Only one small randomized trial directly compared UL and LL SV grafts and reported no significant differences in angiographic or immunohistochemical outcomes.^24^ Subsequent histologic and vasoreactivity studies likewise demonstrated comparable smooth muscle and extracellular matrix characteristics across segments, suggesting that regional biological differences may not translate into clinically meaningful disparities.^25^^,^^26^

In contrast, some reports have suggested potential intrinsic differences between segments. A previous study^27^ reported increased patency of thigh SV grafts and differential expression of remodeling-related proteins, suggesting the possibility of biologic heterogeneity within the vein. Another investigation revealed greater hyperplasia and reduced compliance of LL SVs, which could theoretically predispose grafts to inferior durability.^28^ However, these findings remain inconsistent, are derived from relatively small cohorts, and have not been validated in large clinical populations.

In prior studies from our institution demonstrating the noninferiority of NT-SV grafts relative to arterial grafts,^12^^,^^13^ LL SVs were preferentially harvested, primarily due to concerns regarding size mismatch in composite graft configurations. The present results do not support the assumption that UL SVs confer inferior durability, even when used within contemporary composite graft strategies (Figure 4).

**Figure 4:**
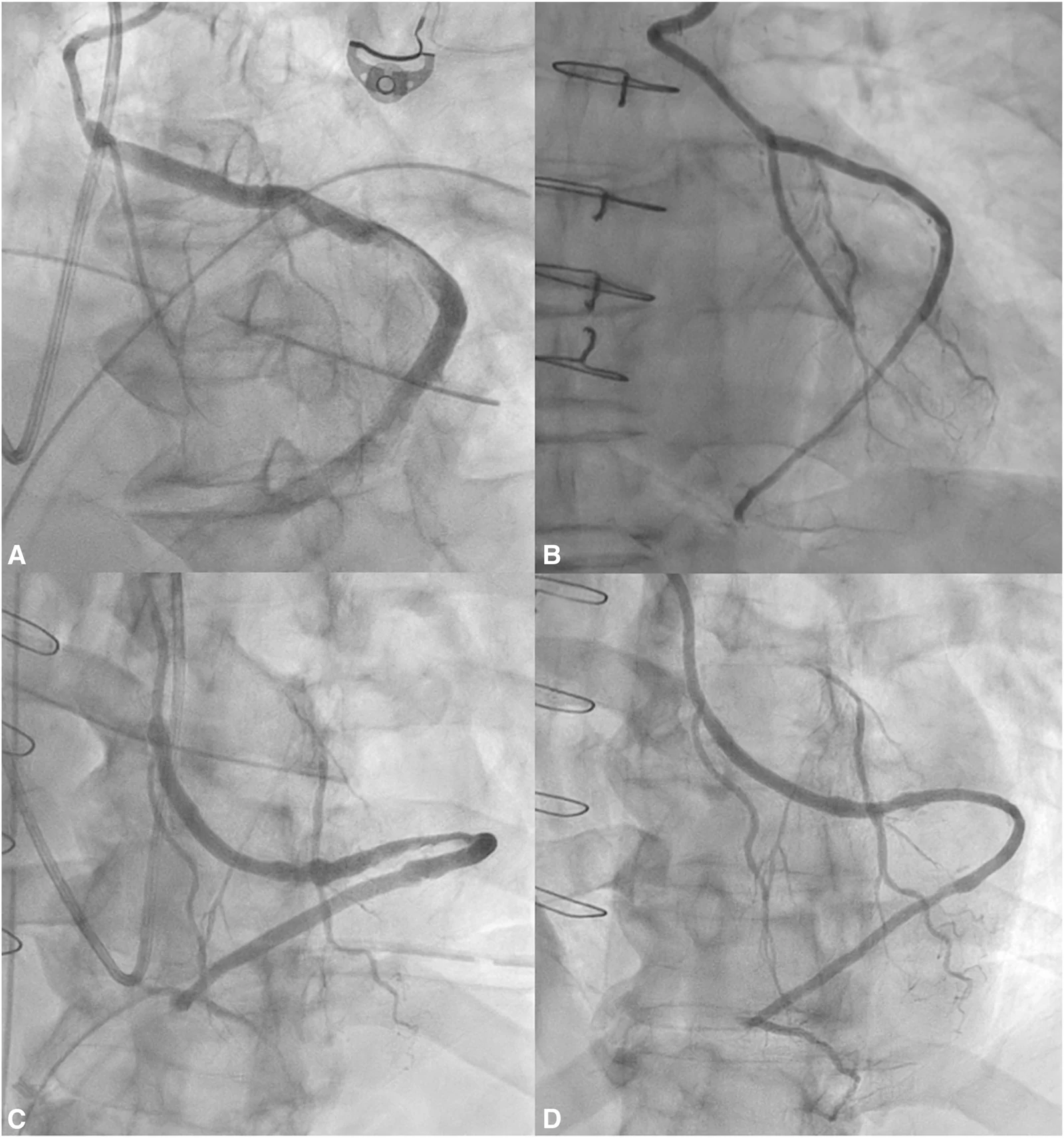
Early and midterm angiograms comparing SV grafts from the upper leg (A and B) versus from the lower leg (C and D). *SV*, Saphenous vein.

LL harvesting may be associated with more pronounced edema, persistent sensory disturbance due to the sacrifice of the saphenous nerve, and wound complications, particularly among patients with limited soft tissue coverage. In contrast, UL harvesting may mitigate these issues and provide greater conduit length, which may facilitate sequential grafting for selected patients.

Given the comparable patency and long-term outcomes across harvest sites, conduit selection may reasonably incorporate considerations of postoperative recovery and patient comfort in addition to graft durability. When anatomically and technically feasible, the preferential use of UL SV may represent a practical strategy to optimize postoperative quality of life without compromising long-term graft performance.

### Study Limitations

There are several limitations to the present study that must be recognized. First, this study was not performed in a prospective randomized manner, although propensity score matching was performed to reduce differences between the groups. Second, 1- and 5-year graft evaluations were not performed in the entire cohort; thus, the potential for overestimated graft patency rates remains. Third, SVs were used as composite grafts based on the ITAs in a substantial portion of the study population, which warrants caution in generalization. Fourth, the variables, including SV quality, SV diameter, or other assessments of target vessels, were not included in the analysis.

## Conclusions

SV grafts from the UL demonstrated comparable 5-year angiographic patency and 7-year clinical outcomes compared with those from the LL.

## Conflict of Interest Statement

The authors reported no conflicts of interest.

The *Journal* policy requires editors and reviewers to disclose conflicts of interest and to decline handling or reviewing manuscripts for which they may have a conflict of interest. The editors and reviewers of this article have no conflicts of interest.

## Figures and Tables

**Figure E1:**
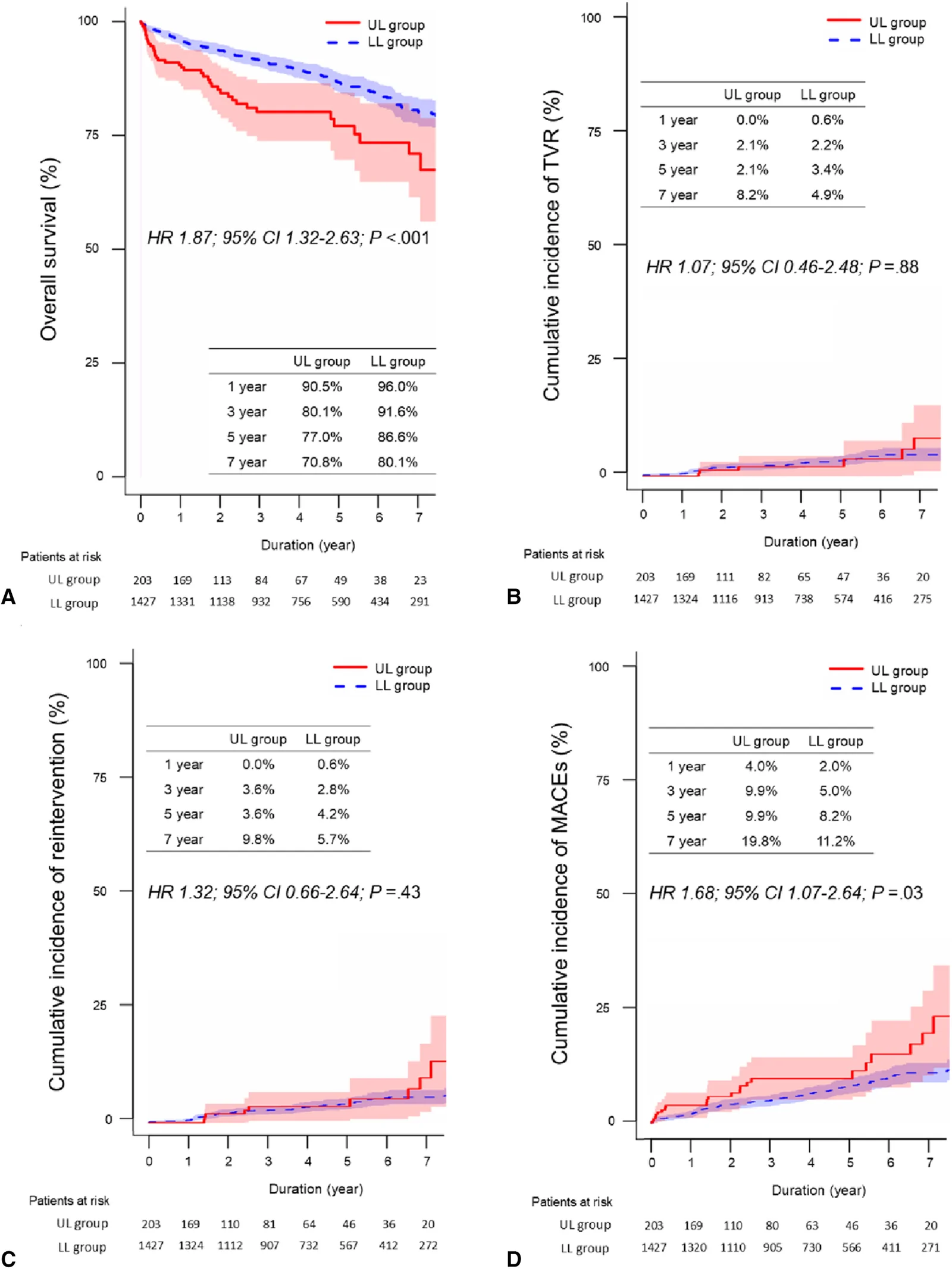
Comparison of (A) overall survival, (B) cumulative incidence of TVR, (C) cumulative incidence of reintervention, and (D) cumulative incidence of MACEs between the groups in the entire population. Shading indicates the 95% CI. *CI*, Confidence interval; *HR*, hazard ratio; *LL*, lower leg; *MACE*, major adverse cardiac event; *TVR*, target vessel revascularization; *UL*, upper leg.

**Table E1:** Comparison of SV graft selection according to the surgeon

Variables	UL group (n = 203)	LL group (n = 1427)	*P*
Surgeon			.92
Surgeon A	164 (12.4%)	1157 (87.6%)	
Surgeon B	39 (12.6%)	270 (87.4%)	

*LL*, Lower leg; *SV*, saphenous vein; *UL*, upper leg.

**Table E2:** The number of patients who underwent follow-up coronary angiograms at each time point, and the reasons for excluding from angiographic follow-up

Variables	Entire population		Matched population	
	UL group (n = 203)	LL group (n = 1427)	UL group (n = 202)	LL group (n = 202)
Early angiography	201 (99.0%)	1421 (99.6%)	201 (99.5%)	200 (99.0%)
Mortality	2 (1.0%)	5 (0.4%)	1 (0.5%)	1 (0.5%)
Refusal	0 (0.0%)	0 (0.0%)	0 (0.0%)	0 (0.0%)
Renal dysfunction	0 (0.0%)	1 (0.1%)	0 (0.0%)	1 (0.5%)
1-y angiography	158 (77.8%)	1246 (87.3%)	158 (78.2%)	166 (82.2%)
Mortality	34 (16.7%)	96 (6.7%)	33 (16.3%)	21 (10.4%)
Refusal	10 (4.9%)	81 (5.7%)	10 (5.0%)	14 (6.9%)
Renal dysfunction	1 (0.5%)	4 (0.3%)	1 (0.5%)	1 (0.5%)
5-y angiography	36 (17.7%)	472 (33.1%)	36 (17.8%)	67 (33.2%)
Mortality	154 (75.9%)	837 (58.7%)	153 (75.7%)	117 (57.9%)
Refusal	13 (6.4%)	118 (8.3%)	13 (6.4%)	18 (8.9%)
Renal dysfunction	0 (0.0%)	0 (0.0%)	0 (0.0%)	0 (0.0%)

*LL*, Lower leg; *UL*, upper leg.

**Table E3:** The number of patients who underwent follow-up coronary angiograms and the percentages of computed tomography angiograms at each time point

Variables	Entire population		Matched population	
	UL group (n = 203)	LL group (n = 1427)	UL group (n = 202)	LL group (n = 202)
Early angiography	201 (99.0%)	1421 (99.6%)	201 (99.5%)	200 (99.0%)
Conventional angiography	200 (99.5%)	1421 (100.0%)	200 (99.5%)	200 (100.0%)
CT angiography	1 (0.5%)	0 (0.0%)	1 (0.5%)	0 (0.0%)
1-y angiography	158 (77.8%)	1246 (87.3%)	158 (78.2%)	166 (82.2%)
Conventional angiography	66 (41.8%)	451 (36.2%)	66 (41.8%)	85 (51.2%)
CT angiography	92 (58.2%)	795 (63.8%)	92 (58.2%)	81 (48.8%)
5-y angiography	36 (17.7%)	472 (33.1%)	36 (17.8%)	67 (33.2%)
Conventional angiography	8 (22.2%)	64 (13.6%)	8 (22.2%)	9 (13.4%)
CT angiography	28 (77.8%)	408 (86.4%)	28 (77.8%)	58 (86.6%)

*CT*, Computed tomography; *LL*, lower leg; *UL*, upper leg.

**Table E4:** Comparison of operative data

Variables	Entire population			Matched population		
	UL group (n = 203)	LL group (n = 1427)	*P*	UL group (n = 202)	LL group (n = 202)	*P*
SV grafting strategy, n (%)			<.001			.214
LITA-based Y-composite grafting	147 (72.4)	1184 (83.0)		146 (72.3)	165 (81.7)	
LITA-based I-composite grafting	14 (6.9)	111 (7.8)		14 (6.9)	16 (7.9)	
RITA-based Y-composite grafting	1 (0.5)	18 (1.3)		1 (0.5)	3 (1.5)	
RITA-based I-composite grafting	9 (4.4)	24 (1.7)		9 (4.5)	1 (0.5)	
Aortocoronary grafting	32 (15.8)	90 (6.3)		32 (15.8)	17 (8.4)	
Number of distal anastomosis per patient						
Per patient	3.55 ± 0.89	3.68 ± 0.98	.12	3.55 ± 0.89	3.66 ± 0.93	.21
Per LITA	0.86 ± 0.39	0.95 ± 0.42	.006	0.86 ± 0.39	0.95 ± 0.42	.02
Per RITA	0.02 ± 0.18	0.02 ± 0.14	.59	0.02 ± 0.18	0.02 ± 0.14	>.99
Per SV	2.67 ± 0.84	2.72 ± 0.91	.51	2.67 ± 0.84	2.69 ± 0.90	.72
Anastomosed to LAD territory	0.83 ± 0.58	0.77 ± 0.56	.20	0.83 ± 0.58	0.77 ± 0.58	.26
Anastomosed to LCX territory	0.97 ± 0.55	1.07 ± 0.63	.03	0.97 ± 0.55	1.04 ± 0.63	.20
Anastomosed to RCA territory	0.87 ± 0.52	0.88 ± 0.60	.91	0.87 ± 0.52	0.88 ± 0.59	.79
As sequential anastomosis	1.65 ± 0.83	1.69 ± 0.90	.55	1.65 ± 0.83	1.66 ± 0.88	.82
As terminal anastomosis	1.02 ± 0.14	1.03 ± 0.17	.66	1.02 ± 0.14	1.03 ± 0.17	.73

*LAD*, Left anterior descending; *LCX*, left circumflex; *LITA*, left internal thoracic artery; *LL*, lower leg; *RCA*, right coronary artery; *RITA*, right internal thoracic artery; *SV*, saphenous vein; *UL*, upper leg.

**Table E5:** Comparison of early, 1-year, and 5-year angiographic patency rates of all anastomoses between the groups

Variables	Entire population			Matched population		
	UL group (n = 203)	LL group (n = 1427)	*P*	UL group (n = 202)	LL group (n = 202)	*P*
Early angiography	(n = 201)	(n = 1421)		(n = 201)	(n = 200)	
Overall	98.7% (704/713)	98.4% (5153/5239)	.445	98.7% (704/713)	98.8% (725/734)	.951
LITA	100.0% (173/173)	99.3% (1340/1349)	.537	100.0% (173/173)	99.5% (190/191)	.044
RITA	100.0% (5/5)	100.0% (23/23)	-	100.0% (5/5)	100.0% (4/4)	-
SV	98.3% (526/535)	98.0% (3790/3867)	.624	98.3% (526/535)	98.5% (531/539)	.795
1-y angiography	(n = 158)	(n = 1246)		(n = 158)	(n = 166)	
Overall	95.2% (534/561)	95.5% (4418/4624)	.722	95.2% (534/561)	96.1% (592/616)	.510
LITA	96.4% (132/137)	97.3% (1153/1185)	.525	96.4% (132/137)	98.1% (157/160)	.397
RITA	100.0% (3/3)	100.0% (20/20)	-	100.0% (3/3)	100.0% (4/4)	-
SV	94.8% (399/421)	94.9% (3245/3419)	.916	94.8% (399/421)	95.4% (431/452)	.736
5-y angiography	(n = 36)	(n = 472)		(n = 36)	(n = 67)	
Overall	98.4% (123/125)	94.5% (1605/1699)	.069	98.4% (123/125)	96.7% (236/244)	.343
LITA	100.0% (32/32)	96.4% (449/466)	.527	100.0% (32/32)	98.5% (67/68)	.283
RITA	- (0/0)	100.0% (2/2)	-	- (0/0)	- (0/0)	-
SV	97.8% (91/93)	93.7% (1154/1231)	.118	97.8% (91/93)	96.0% (169/176)	.425

*LITA*, Left internal thoracic artery; *LL*, lower leg; *RITA*, right internal thoracic artery; *SV*, saphenous vein; *UL*, upper leg.

**Table E6:** Risk factor analysis for overall survival

Variables	Univariate analysis			Multivariate analysis		
	*P*	HR	95% CI	*P*	HR	95% CI
Age, y						
≤65	.59	1.01	0.98-1.04	.41	1.01	0.98-1.05
>65	<.001	1.08	1.06-1.10	<.001	1.08	1.06-1.11
Sex, female	.54	0.91	0.68-1.22			
Risk factors						
Smoking	.04	0.75	0.57-0.99	.50	0.91	0.68-1.21
Body mass index	<.001	0.87	0.83-0.91	<.001	0.91	0.87-0.95
Hypertension	.12	1.26	0.94-1.69			
Diabetes mellitus	.39	1.12	0.87-1.45			
Dyslipidemia	.03	0.75	0.57-0.98	.44	0.90	0.68-1.18
History of stroke	.10	1.33	0.95-1.87	.75	0.95	0.67-1.34
Dialysis	<.001	3.48	2.50-4.84	<.001	4.45	3.12-6.34
Chronic obstructive pulmonary disease	<.001	2.70	1.76-4.16	.003	1.98	1.26-3.12
Atrial fibrillation	.02	2.17	1.12-4.23	.35	1.39	0.70-2.78
Peripheral vascular disease	.77	1.07	0.68-1.68			
Previous percutaneous coronary intervention	.28	0.84	0.61-1.16			
Left ventricular ejection fraction ≤0.35	<.001	2.57	1.91-3.46	<.001	2.02	1.49-2.76
Acute coronary syndrome	.18	1.20	0.92-1.57			
Three vessel disease	.24	1.20	0.89-1.63			
Left main disease	.87	0.98	0.75-1.27			
Emergency operation	<.001	2.32	1.66-3.24	.002	1.72	1.22-2.42
Surgeon B∗	<.001	0.30	0.16-0.55	<.001	0.29	0.16-0.53
SV graft selection†	<.001	1.87	1.32-2.63	.11	1.33	0.94-1.89

*CI*, Confidence interval; *HR*, hazard ratio; *SV*, saphenous vein.

∗ Surgeon A.

† Lower leg.

**Table E7:** Risk factor analysis for target vessel revascularization

Variables	Univariate analysis			Multivariate analysis		
	*P*	HR	95% CI	*P*	HR	95% CI
Age, y	.60	0.99	0.97-1.02			
Sex, female	.22	0.70	0.40-1.23			
Risk factors						
Smoking	.99	1.00	0.58-1.72			
Body mass index	.47	0.98	0.92-1.04			
Hypertension	.80	0.93	0.53-1.64			
Diabetes mellitus	.49	1.20	0.71-2.05			
Dyslipidemia	.77	1.08	0.64-1.84	.11	0.38	0.12-1.24
History of stroke	.09	0.36	0.11-1.16			
Dialysis	.69	1.20	0.48-3.01			
Chronic obstructive pulmonary disease	.34	0.38	0.05-2.79			
Atrial fibrillation	.54	0.42	0.00-2.88			
Peripheral vascular disease	.91	1.06	0.42-2.65			
Previous percutaneous coronary intervention	.09	1.63	0.92-2.87	.09	1.64	0.92-2.94
Left ventricular ejection fraction ≤0.35	.04	0.12	0.02-0.90	.04	0.12	0.02-0.89
Acute coronary syndrome	.56	0.85	0.50-1.46			
Three vessel disease	.26	0.73	0.42-1.27			
Left main disease	.83	0.94	0.54-1.63			
Emergency operation	.68	1.20	0.51-2.82			
Surgeon B∗	.60	0.79	0.33-1.90	.48	0.72	0.30-1.75
SV graft selection†	.88	1.07	0.46-2.48	.72	1.17	0.50-2.75

*CI*, Condifence interval; *HR*, hazard ratio; *SV*, saphenous vein.

∗ Surgeon A.

† Lower leg.

**Table E8:** Risk factor analysis for reintervention

Variables	Univariate analysis			Multivariate analysis		
	*P*	HR	95% CI	*P*	HR	95% CI
Age, y	.66	1.00	0.97-1.02			
Sex, female	.21	0.72	0.43-1.20			
Risk factors						
Smoking	.90	0.97	0.60-1.57			
Body mass index	.42	0.97	0.92-1.04			
Hypertension	.67	0.90	0.54-1.48			
Diabetes mellitus						
At 1 y	.06	0.57	0.32-1.03	.09	0.59	0.32-1.08
At 5 y	.06	1.88	0.98-3.62	.04	1.96	1.03-3.75
Dyslipidemia	.76	1.08	0.67-1.73			
History of stroke	.60	0.82	0.39-1.71			
Dialysis	.44	1.36	0.62-2.96			
Chronic obstructive pulmonary disease	.64	1.27	0.47-3.47			
Atrial fibrillation	.44	0.33	0.00-2.25			
Peripheral vascular disease	.99	1.00	0.44-2.32			
Previous percutaneous coronary intervention	.07	1.59	0.96-2.65	.08	1.60	0.95-2.69
Left ventricular ejection fraction ≤0.35	.04	0.30	0.09-0.97	.04	0.30	0.09-0.96
Acute coronary syndrome	.84	1.05	0.65-1.70			
Three vessel disease	.06	0.63	0.39-1.02	.12	0.68	0.41-1.10
Left main disease	.88	1.04	0.64-1.67			
Emergency operation	.30	1.45	0.72-2.95			
Surgeon B∗	.45	0.74	0.33-1.63	.34	0.68	0.30-1.51
SV graft selection†	.43	1.32	0.66-2.64	.38	1.36	0.68-2.72

*CI*, Confidence interval; *HR*, hazard ratio; *SV*, saphenous vein.

∗ Surgeon A.

† Lower leg.

**Table E9:** Risk factor analysis for major adverse cardiac events

Variables	Univariate analysis			Multivariate analysis		
	*P*	HR	95% CI	*P*	HR	95% CI
Age, y	.25	1.01	0.99-1.03			
Sex, female	.20	0.79	0.54-1.14			
Risk factors						
Smoking	.33	0.84	0.59-1.19			
Body mass index	.01	0.93	0.88-0.99	.09	0.95	0.90-1.01
Hypertension	.26	1.24	0.85-1.82			
Diabetes mellitus	.18	1.26	0.90-1.76			
Dyslipidemia	.65	0.92	0.66-1.30			
History of stroke	.99	1.00	0.62-1.61			
Dialysis	<.001	2.40	1.52-3.80	.003	2.08	1.27-3.39
Chronic obstructive pulmonary disease	.05	1.85	1.01-3.41	.22	1.49	0.79-2.82
Atrial fibrillation	.17	1.92	0.67-4.83			
Peripheral vascular disease	.34	0.72	0.37-1.41			
Previous percutaneous coronary intervention	.05	1.44	0.99-2.08	.05	1.46	1.00-2.12
Left ventricular ejection fraction ≤0.35						
At 1 y	<.001	2.56	1.70-3.86	.004	1.90	1.23-2.94
At 5 y	.27	1.37	0.79-2.38	.66	1.14	0.64-2.04
Acute coronary syndrome	.03	1.50	1.05-2.14	.21	1.27	0.87-1.84
Three vessel disease	.92	1.02	0.70-1.48			
Left main disease	.85	0.97	0.69-1.36			
Emergency operation						
At 1 y	.03	1.77	1.06-2.97	.26	1.37	0.79-2.34
At 5 y	.29	0.66	0.31-1.43	.17	0.58	0.26-1.28
Surgeon B∗	.03	0.48	0.25-0.92	.03	0.49	0.25-0.93
SV graft selection†	.03	1.68	1.07-2.64	.16	1.39	0.88-2.19

*CI*, Confidence interval; *HR*, hazard ratio; *SV*, saphenous vein.

∗ Surgeon A.

† Lower leg.

## References

[1] Cameron A., Davis K.B., Green G., Schaff H.V. (1996). Coronary bypass surgery with internal-thoracic-artery grafts--effects on survival over a 15-year period. N Engl J Med.

[2] Volk L., Cornwell L., Pajaro O. (2026). Arterial graft choice and outcomes within the Veterans Administration: a 20-year review of 47,525 patients. J Thorac Cardiovasc Surg.

[3] Dayan V., Hernandez M., Montero J.A., Sosa C., Cubas S., Bakaeen F. (2026). Long-term outcomes of patients undergoing multiarterial grafting in coronary artery bypass grafting: insights from a Latin American national cohort. J Thorac Cardiovasc Surg.

[4] Gaudino M., Chikwe J., Falk V., Lawton J.S., Puskas J.D., Taggart D.P. (2020). Transatlantic editorial: the use of multiple arterial grafts for coronary revascularization in Europe and North America. J Thorac Cardiovasc Surg.

[5] Goldman S., Zadina K., Moritz T. (2004). Long-term patency of saphenous vein and left internal mammary artery grafts after coronary artery bypass surgery: results from a Department of Veterans Affairs Cooperative Study. J Am Coll Cardiol.

[6] Magee M.J., Alexander J.H., Hafley G. (2008). Coronary artery bypass graft failure after on-pump and off-pump coronary artery bypass: findings from PREVENT IV. Ann Thorac Surg.

[7] Souza D. (1996). A new no-touch preparation technique. Technical notes. Scand J Thorac Cardiovasc Surg.

[8] Souza D.S., Johansson B., Bojo L. (2006). Harvesting the saphenous vein with surrounding tissue for CABG provides long-term graft patency comparable to the left internal thoracic artery: results of a randomized longitudinal trial. J Thorac Cardiovasc Surg.

[9] Samano N., Geijer H., Liden M., Fremes S., Bodin L., Souza D. (2015). The no-touch saphenous vein for coronary artery bypass grafting maintains a patency, after 16 years, comparable to the left internal thoracic artery: a randomized trial. J Thorac Cardiovasc Surg.

[10] Verma S., Lovren F., Pan Y. (2014). Pedicled no-touch saphenous vein graft harvest limits vascular smooth muscle cell activation: the PATENT saphenous vein graft study. Eur J Cardiothorac Surg.

[11] Dreifaldt M., Mannion J.D., Geijer H., Lidén M., Bodin L., Souza D. (2021). The no-touch saphenous vein is an excellent alternative conduit to the radial artery 8 years after coronary artery bypass grafting: a randomized trial. J Thorac Cardiovasc Surg.

[12] Hwang H.Y., Lee Y., Sohn S.H., Choi J.W., Kim K.B. (2021). Equivalent 10-year angiographic and long-term clinical outcomes with saphenous vein composite grafts and arterial composite grafts. J Thorac Cardiovasc Surg.

[13] Kim M.S., Hwang H.Y., Kim J.S., Oh S.J., Jang M.J., Kim K.B. (2018). Saphenous vein versus right internal thoracic artery as a Y-composite graft: Five-year angiographic and clinical results of a randomized trial. J Thorac Cardiovasc Surg.

[14] Sohn S.H., Kang Y., Kim J.S., Choi J.W., Hwang H.Y. (2024). The impact of perivascular tissue preservation on 5-year patency of saphenous vein composite grafts. Interdiscip Cardiovasc Thorac Surg.

[15] Kim K.B., Choi J.W., Oh S.J. (2020). Twenty-year experience with off-pump coronary artery bypass grafting and early postoperative angiography. Ann Thorac Surg.

[16] FitzGibbon G.M., Burton J.R., Leach A.J. (1978). Coronary bypass graft fate: angiographic grading of 1400 consecutive grafts early after operation and of 1132 after one year. Circulation.

[17] Heinze G., Schemper M. (2002). A solution to the problem of separation in logistic regression. Stat Med.

[18] Mondol M.H., Rahman M.S. (2019). Bias-reduced and separation-proof GEE with small or sparse longitudinal binary data. Stat Med.

[19] Austin P.C., Fine J.P. (2019). Propensity-score matching with competing risks in survival analysis. Stat Med.

[20] Gaudino M., Benedetto U., Fremes S. (2018). Radial-artery or saphenous-vein grafts in coronary-artery bypass surgery. N Engl J Med.

[21] Gaudino M., Benedetto U., Fremes S. (2020). Association of radial artery graft vs saphenous vein graft with long-term cardiovascular outcomes among patients undergoing coronary artery bypass grafting: a systematic review and meta-analysis. JAMA.

[22] Buxton B.F., Hayward P.A., Raman J. (2020). Long-term results of the RAPCO trials. Circulation.

[23] Taggart D.P., Benedetto U., Gerry S. (2019). Bilateral versus single internal-thoracic-artery grafts at 10 years. N Engl J Med.

[24] Chang H.W., Hwang H.Y., Seo J.W., Kim K.B. (2018). Lower versus upper leg saphenous vein composite grafts based on the left internal thoracic artery: a randomized study. J Thorac Cardiovasc Surg.

[25] Stooker W., Gök M., Sipkema P. (2003). Pressure-diameter relationship in the human greater saphenous vein. Ann Thorac Surg.

[26] Golbasi I., Tasatargil A., Aksoy N.H. (2005). A functional and histopathological comparison of proximal and distal saphenous vein contractility and morphology. Tex Heart Inst J.

[27] Chen A., Pan K. (2025). The thigh saphenous vein versus the calf saphenous vein: searching for the optimal conduit for coronary artery bypass grafting. Catheter Cardiovasc Interv.

[28] Davies A.H., Magee T.R., Baird R.N., Sheffield E., Horrocks M. (1992). Vein compliance: a preoperative indicator of vein morphology and of veins at risk of vascular graft stenosis. Br J Surg.

